# Optimizing rhizosphere metabolic circular economy toward sustainable ecosystems

**DOI:** 10.1093/sumbio/qvag037

**Published:** 2026-08-27

**Authors:** Kunkun Fan, Zhihui Xu, Kai Sun, Haiyan Chu

**Affiliations:** State Key Laboratory of Soil and Sustainable Agriculture, Institute of Soil Science, Chinese Academy of Sciences, Nanjing 211135, China; College of Advanced Agricultural Sciences, University of Chinese Academy of Sciences, Beijing 101408, China; Jiangsu Provincial Key Lab for Organic-based Fertilizers Creation and Soil Health Manipulation, Jiangsu Collaborative Innovation Center for Solid Organic Wastes, Educational Ministry Engineering Center of Resource-Saving Fertilizers, Nanjing Agricultural University, Nanjing 211800, China; Jiangsu Key Laboratory for Pathogens and Ecosystems, Jiangsu Engineering and Technology Research Center for Industrialization of Microbial Resources, College of Life Sciences, Nanjing Normal University, Nanjing 210023, China; State Key Laboratory of Soil and Sustainable Agriculture, Institute of Soil Science, Chinese Academy of Sciences, Nanjing 211135, China; College of Advanced Agricultural Sciences, University of Chinese Academy of Sciences, Beijing 101408, China

**Keywords:** rhizosphere, metabolic circular economy, plant−microbe interactions, microbial necromass, sustainable ecosystems

## Abstract

The rhizosphere metabolic circular economy (RMCE) governs the exchange, reuse, and reconfiguration of metabolites at the root−soil interface. Building on traditional binary plant−microbe models, this perspective advances an expanded RMCE framework by integrating three additional interdependent dimensions that explain how rhizosphere metabolic processes persist over time, organize across space, and recover under environmental stress. First, the live-dead plant−microbe continuum enables metabolic persistence by incorporating microbial necromass and root residues into long-term carbon sequestration and nutrient regeneration. Second, spatial organization across rhizosphere microzones optimizes microscale metabolic interactions through niche heterogeneity. Third, adaptive restoration dynamically re-establishes disrupted metabolic networks through coordinated plant−microbiome stress responses. We further outline scenario-based management strategies to translate the RMCE insights into practical solutions, which necessitates multiple approaches targeting root exudate quality, microbial necromass dynamics, rhizosphere spatial architecture, and cross-kingdom signaling. Together, these advances redefine RMCE as a dynamically regulated, spatially structured, and ecologically complex system, providing a more comprehensive basis for its optimization in sustainable ecosystems.

**Sustainability Statement**

This article primarily aligns with UN Sustainable Development Goal (SDG) 15 (Life on Land), as it focuses on optimizing rhizosphere metabolic circular economy (RMCE) to restore soil health, enhance biodiversity, and promote sustainable ecosystem functioning. The proposed framework also directly supports SDG 2 (Zero Hunger) and SDG 13 (Climate Action) by advancing agricultural practices that improve crop resilience and nutrient efficiency under climate stress. The RMCE insights provide a pathway for developing resilient ecosystems that balance productivity with long-term environmental sustainability.

## Introduction

The rhizosphere, a highly dynamic soil microzone at the root−soil interface, is crucial for plant−microbe interactions and biogeochemical cycling, functioning as a hotspot of labile organic matter turnover and metabolic activity (Mueller et al. 2024). Over >450 million years of coevolution, plants and rhizosphere microorganisms have developed intricate reciprocal exchanges, conceptualized as the *Rhizosphere Metabolic Circular Economy* (RMCE), a nearly “waste-free” network governing the exchange, reuse, and reconfiguration of metabolites (Korenblum et al. 2022). Plants invest *c*. 20%−40% of photosynthetic carbon into root exudates to selectively shape microbial communities by recruiting beneficial taxa, and suppressing pathogens (Zhalnina et al. 2018, Korenblum et al. 2022). In return, microorganisms metabolize these exudates to mobilize recalcitrant soil nutrients and produce bioactive metabolites that influence plant growth, defense, and nutrient acquisition (Korenblum et al. 2022). The RMCE, therefore underpins soil fertility, plant resilience, and the regulation of terrestrial biogeochemical cycles.

However, RMCE is disrupted by soil degradation (e.g. acidification, salinization, alkalinization, compaction, nutrient imbalances, and reduced microbial diversity), and the prevalence of soil-borne diseases (Pathak et al. 2024), challenges that are critically exacerbated by the accelerating pressures of global climate change and intensive agricultural practices. For example, acidic soils, covering *c*. 40% of global farmland, can induce aluminum (Al) toxicity, thereby disrupting root cell walls and nutrient cycling (Che et al. 2023). Continuous cropping further impairs the RMCE by accumulating autotoxic metabolites that hinder rhizosphere metabolic recycling and shift microbiome toward pathogenic dominance (Chen et al. 2023). Moreover, excessive nitrogen fertilization inhibits arbuscular mycorrhizal (AM) fungi colonization, reducing phosphorus (P) cycling efficiency (Abbasi 2023). Together, these disruptions compromise soil health, undermine plant resilience, and exacerbate environmental pressures (e.g. nutrient leaching and greenhouse gas emissions). Optimizing the RMCE is therefore an urgent priority for achieving sustainable ecosystem management.

**Figure 1:**
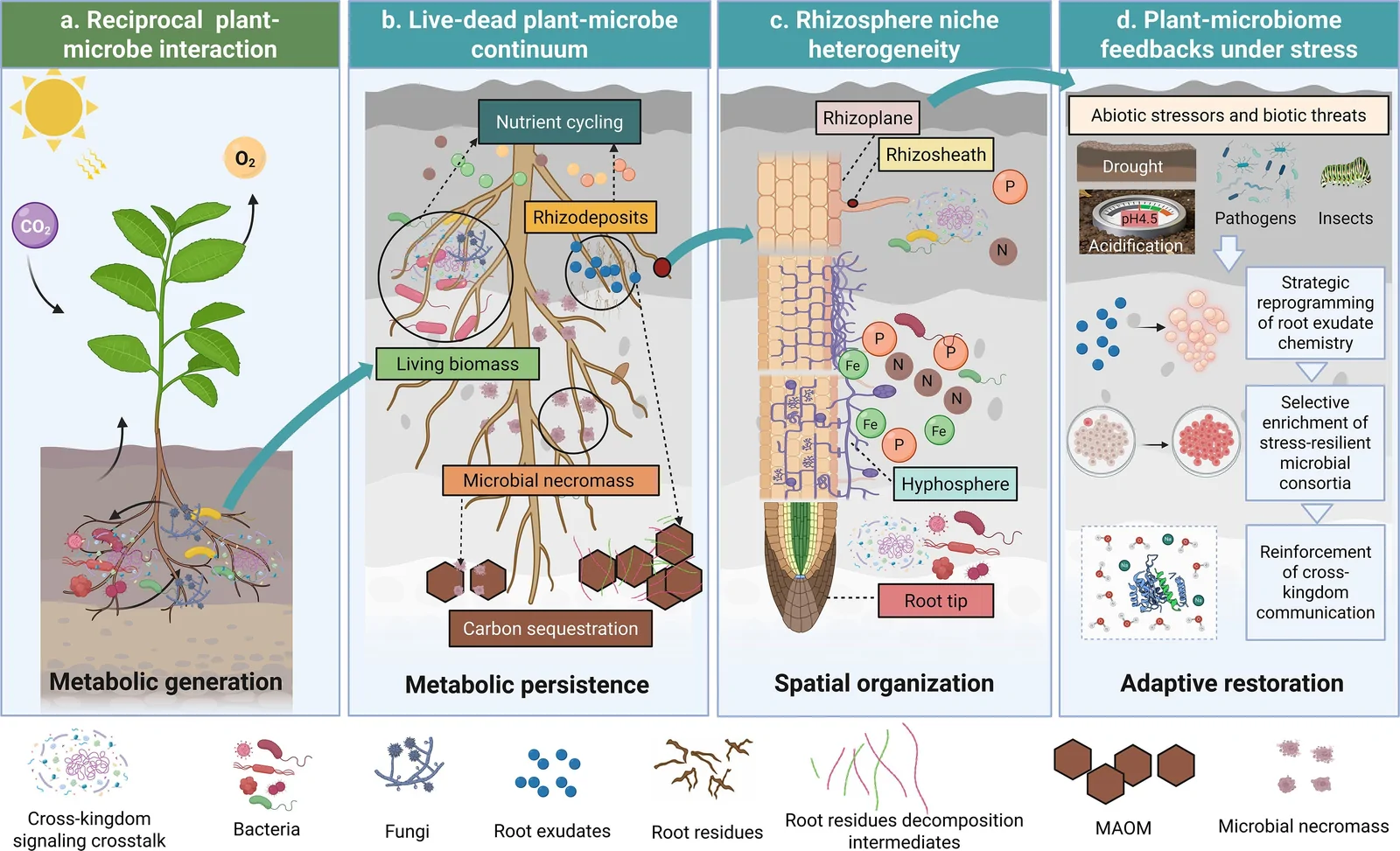
Conceptual diagram of the expanded RMCE framework. This figure was created using BioRender (biorender.com).

Existing RMCE frameworks largely emphasize binary plant−microbe interactions (Korenblum et al. 2022). However, three critical components remain underrepresented: (i) the live-dead plant−microbe continuum, where microbial necromass and dead fine roots act as pivotal carriers for long-term carbon and nutrient cycling; (ii) rhizosphere spatial heterogeneity (e.g. rhizoplane, rhizosheath, and hyphosphere), which creates distinct microniches for specialized physical, biological, and chemical processes; and (iii) coordinated adaptive responses of plants and microorganisms to environmental stress, which serve as dynamic repair mechanisms. In this perspective, we propose that RMCE emerges from four sequential yet tightly interconnected ecological processes: (i) metabolic generation through reciprocal plant−microbe interactions; (ii) metabolic persistence across the live-dead continuum; (iii) spatial organization via rhizosphere niche differentiation; and (iv) adaptive restoration mediated by stress-responsive plant−microbiome feedbacks. Together, these processes establish a reinforcing RMCE framework that enables rhizosphere systems to maintain function, recover from disturbance, and adapt to environmental change (Fig. 1). By explicitly linking plant and microbial traits with soil processes and scenario-based management, we aim to translate the RMCE insights into scalable solutions for sustainable agriculture and ecosystem resilience.

## Advancing the RMCE framework

### Reciprocal plant−microbe metabolism generates RMCE

Reciprocal metabolic exchange between plants and microorganisms constitutes the structural and functional core of the RMCE (Fig. 1a) (Korenblum et al. 2022). Host plants secrete a diverse array of metabolites, including primary metabolites (e.g. sugars, amino acids, organic acids) serving as carbon and nitrogen sources for microbial growth, and secondary metabolites (e.g. flavonoids, coumarins, benzoxazinoids, terpenes) priming symbiosis, suppressing pathogens, and selectively assembling microbiomes (Hu et al. 2018, Zhalnina et al. 2018). Recent evidence highlights the specificity and dynamism of these chemical dialogues. For instance, localized and transient leakages of amino acids, particularly glutamine from the vasculature, create highly favorable metabolic niches that attract bacteria and drive their colonization and proliferation (Tsai et al. 2025). In maize (*Zea mays*), benzoxazinoid exudates (e.g. DIMBOA, MBOA, and related benzoxazinoids) shape the composition of the root-associated microbiota, including plant growth-promoting rhizobacteria such as *Pseudomonas putida*, thereby contributing to systemic defense priming and enhanced resistance against pests and pathogens (Hu et al. 2018). These exudates are highly dynamic, varying with plant species, developmental stage, and environmental conditions.

In return, microorganisms transform plant-derived exudates to drive functions that reinforce RMCE circularity. Typically, microorganisms solubilize recalcitrant Fe via siderophores; mobilize P through the secretion of organic acids or modulating phosphatase activity; promote plant growth by synthesizing phytohormones (e.g. indole-3-acetic acid, gibberellins); concurrently, modulate plant ethylene (ETH) signaling through the production of 1-aminocyclopropane-1-carboxylate (ACC) deaminase, thereby mitigating growth inhibition under abiotic stresses. Beyond direct interactions, microbial cross-feeding networks further enhance the efficiency of RMCE. For example, complex substrates such as poly-γ-glutamic acid can mediate synergistic interactions among microbial taxa, linking carbon processing with nutrient mobilization and reinforcing resource cycling in the rhizosphere (Gu et al. 2025).

Advances in rhizosphere ecology over the past decade reveal that plant−microbe communication extends far beyond nutrient exchange. Multitiered signaling mediated by root exudates, microbial metabolites, quorum sensing molecules, volatile organic compounds, and peptide signals dynamically regulates microbial community assembly and host physiology (Korenblum et al. 2022). Quorum sensing (QS) enables bidirectional communication, with bacterial N-acyl homoserine lactones (AHLs) directly influencing gene expression, hormone signaling, and developmental processes in plants (Ortiz-Castro and López-Bucio 2019). Host plants have evolved sophisticated strategies to perceive and respond to these bacterial AHLs, whereby short-chain AHLs promote plant growth and root elongation, whereas long-chain variants prime plant immune defenses (Shrestha et al. 2022). In addition to AHLs, cyclodipeptides (CDPs) have emerged as an important class of bacterial QS molecules that modulate bacterial communication and biofilm formation, while also influencing plant growth and immune responses (Ortiz-Castro et al. 2011). Conversely, plant-derived metabolites such as p-coumaric acid are exploited by specific microorganisms as substrates for the biosynthesis of modified AHL signals, fine-tuning quorum-sensing-regulated behaviors within the rhizosphere microbial populations (Schaefer et al. 2008).

These reciprocal interactions largely describe rapid metabolic fluxes among living organisms. However, they alone cannot explain how rhizosphere metabolic functions persist beyond the lifespan of individual organisms. We therefore propose that RMCE extends across a live-dead continuum, whereby metabolites and nutrients continue to circulate through microbial necromass, root residues, and stabilized soil organic matter.

### Metabolic persistence across the live-dead continuum

While living entities drive rapid metabolic exchange, long-term RMCE depends on the persistence of metabolic functions across the live-dead plant−microbe continuum (Fig. 1b). This continuum integrates living biomass, microbial necromass, rhizodeposition, and plant residues into the iterative cycling of carbon, nutrients, and metabolites. Compared to aboveground inputs, root-derived carbon is stabilized far more efficiently, driving mineral-associated organic matter (MAOM) formation at rates 2.4−12 times higher than those of leaf litter (Sokol and Bradford 2019).

Microbial necromass accounts for roughly half of global soil organic carbon (Wang et al. 2021). Its long-term persistence stems from distinct biochemical composition and decomposition dynamics. For example, fungal necromass, rich in chitin, decomposes up to 40% slower than bacterial necromass (Sokol and Bradford 2019). The decomposition process itself is enzymatically driven, where chitinase and glucanases break down fungal cell walls, while proteases and peptidoglycan hydrolases target bacterial residues (Salas et al. 2024). Fine root senescence supplies both labile and recalcitrant substrates, which are decomposed by microbial enzymes (e.g. laccases, peroxidases, cellulases) and re-assimilated into plant−microbe metabolic cycles within the rhizosphere (Loeppmann et al. 2016). This establishes a direct feedback loop between plant carbon allocation and microbial processing. Management practices that promote microbial biomass production and necromass protection, including functionally selected microbial consortia, residue retention, and reduced tillage, can enhance long-term carbon sequestration while maintaining nutrient availability. These processes are effectively integrated in intercropping systems, where plant diversity boosts microbial necromass accumulation through complementary root traits and exudation patterns (Zhu et al. 2024). Spatially, this continuum differs significantly between rhizosphere and bulk soils, with rhizosphere soils showing 45%−646% higher microbial necromass carbon regardless of plant functional group or season (Lu et al. 2023). This enrichment creates localized carbon hotspots, where microbial metabolic traits regulate necromass accumulation differently in absorptive versus transport roots (Wang et al. 2024).

The ultimate fate of carbon within this continuum depends on its transformation into MAOM through microbial processing. Microbial carbon use efficiency (CUE) plays a pivotal role in determining whether carbon flows toward stable MAOM or remains as transient particulate organic matter (Li et al. 2024). High CUE in low-carbon soils promotes rapid necromass production and MAOM formation, while low CUE in high-carbon soils limits additional stabilization capacity (Li et al. 2024). This dynamic explains why belowground inputs create more stable carbon despite their apparent lability—their immediate microbial processing in mineral-rich environments facilitates direct incorporation into protected pools (Sokol and Bradford 2019). The resilience of this continuum against environmental fluctuations emerges from this multilayered protection system, where physical occlusion within aggregates and chemical binding to mineral surfaces synergize with microbial community composition to maintain long-term carbon storage (Hu et al. 2024).

Collectively, these processes enable metabolic functions initiated by living organisms to persist beyond organismal turnover, sustaining rhizosphere carbon cycling and nutrient regeneration over ecological timescales. However, necromass formation and stabilization are highly heterogeneous processes dependent on where microorganisms colonize, die, and interact, this live-dead continuum and its resultant slow-cycling resource pools cannot be fully understood without integrating the spatial organization of rhizosphere metabolic processes.

### Spatial organization through rhizosphere niche heterogeneity

The rhizosphere is not a uniform zone but a highly structured, spatially heterogeneous microenvironment (Fig. 1c). Distinct subzones, including the rhizoplane, rhizosheath, and hyphosphere differ fundamentally in their physical structure, redox potential, nutrient gradients, and metabolite profiles (Mueller et al. 2024). This spatial complexity is not incidental; rather, it is a core driver of RMCE efficiency, enabling microscale niche complementarity. By specializing in different zones, plants and microorganisms reduce direct competition, enable parallel processing of diverse substrates, and create streamlined, efficient resource pathways.

Plants actively engineer this spatial organization. Root tips typically release sugars to prime microbial colonization, while mature root zones secrete aromatic organic acids to mobilize recalcitrant nutrients (Zhalnina et al. 2018). Microbial guilds align with these gradients: copiotrophic bacteria dominate the rhizoplane, acting as a “rapid service hub” for immediate nutrient solubilization, while oligotrophs populate the rhizosheath, functioning as a “slow-release nutrient bank” for decomposing necromass (Mo et al. 2022). This organization is further extended by the hyphosphere through plant−fungus−bacterium interactions (Duan et al. 2024). In the hyphosphere, a diverse community of “hyphospheric bacteria” feeds on fungal metabolites that contain plant-derived carbon. In return, these bacteria enhance fungal nutrition by secreting extracellular enzymes that mineralize complex nutrients, making them accessible to diverse fungal hosts, including AM and ectomycorrhizal fungince. This reciprocal exchange establishes a functional continuum (Duan et al. 2024): carbon flows from the plant to mycorrhizal fungi and onward to hyphospheric bacteria, while mineral nutrients, particularly phosphorus, nitrogen, and iron, are channeled back from bacterial and fungal networks to the host plant. Concurrently, soil aggregates add another layer of spatial complexity by generating microsites with distinct redox conditions, enabling simultaneous processes such as nitrification and denitrification (Philippot et al. 2024).

Importantly, this spatial complexity is a direct target for precision management. Practices that homogenize the rhizosphere, such as soil compaction, uniform broadcast fertilization, or monocropping, erode niche complementarity (Wang et al. 2020), undermining RMCE efficiency. Conversely, interventions that restore or enhance spatial heterogeneity can reboot RMCE efficiency. Crop diversification with varied root architectures and exudate profiles can increase niche heterogeneity and complementarity, thereby supporting the coexistence of functionally distinct microbial guilds (Wang and Kuzyakov 2023). Similarly, the precision placement of fertilizers or amendments can stimulate targeted microbial guilds where they are most effective (Wang and Kuzyakov 2023), while reduced tillage preserves soil aggregate structures and their associated metabolite gradients (Shang et al. 2023). Rather than simply increasing biodiversity, spatial heterogeneity organizes complementary microbial functions by reducing niche overlap, facilitating metabolic cross-feeding, and enhancing the efficiency of resource utilization. However, this spatially organized metabolic network is continuously exposed to environmental fluctuations (e.g. drought, salinity, soil acidification, and pathogen invasion), RMCE inherently requires adaptive mechanisms capable of restoring disrupted metabolic functions.

### Adaptive restoration via stress-responsive plant−microbiome feedbacks

Environmental perturbations continuously disrupt rhizosphere metabolic circularity. We propose that RMCE is restored through adaptive plant−microbiome feedbacks, whereby plants dynamically reprogram root exudation, recruit stress-resilient microbial partners, and reinforce cross-kingdom signaling to re-establish metabolic exchange (Fig. 1d). In the context of global climate change, abiotic stressors such as drought, soil acidification, and salinity, along with biotic threats like pathogen invasion, directly and continuously compromise the integrity of RMCE. RMCE resilience depends on a suite of integrated and adaptive responses, involving strategic reprogramming of root exudate chemistry, selective enrichment of stress-resilient microbial consortia, and reinforcement of cross-kingdom communication.

Drought, one of the most pervasive abiotic stresses, threatens RMCE by disrupting root−microbe interactions, suppressing microbial activity, and limiting nutrient cycling, ultimately reducing plant productivity and ecosystem stability (Pantigoso et al. 2025). During drought, plants reduce total root exudation by 40%−60% and reprogram exudate composition toward signaling molecules, including phytohormones (e.g. ABA, ETH, JA), small molecules (e.g. strigolactones, nitric oxide), and peptides (e.g. CLE peptides), which regulate microbial community assembly and function (Jiang et al. 2023). Strigolactones enhance AM fungi colonization and stimulate drought-responsive traits in rhizobacteria (Chandrasekaran et al. 2025), while nitric oxide mediates microbial recruitment and modulates plant ETH signaling (Lubyanova et al. 2022). CLE peptides secreted by plant roots under drought conditions can interact with microbial receptors to trigger the production of growth regulators that alleviate drought stress. Concurrently, plants shift from sugar to organic acid exudation (e.g. citrate, malate), recruiting beneficial microbes that sustain phosphorus and nitrogen cycling under low water availability (Sorty et al. 2025). Importantly, this shift also enriches ACC deaminase-producing bacteria, which degrade the ETH precursor ACC, thereby reducing stress-induced ETH accumulation (Moon and Ali 2022). This alleviates root growth inhibition and supports continued root development under drought.

In acidic soils, Al^3+^ toxicity threatens RMCE by binding to root cell wall pectins and plasma membranes, causing structural damage and impairing nutrient uptake. Plants counter this stress through a dual strategy, secreting phenolic acids (e.g. caffeic and ferulic acid) that chelate Al^3+^ into nontoxic complexes, and simultaneously reshaping the rhizosphere microbiome to enrich Al-tolerant bacterial taxa (Ling et al. 2023). Evidence from *Pinus massoniana* under Al stress shows that shifts in root exudate profiles are closely associated with the recruitment of microbial communities that enhance antioxidant enzyme activities (*e.g*. SOD, POD) and stabilize root architecture (Ling et al. 2023). While direct evidence for microbial secretion of xyloglucan endotransglucosylase remains limited, it is plausible that recruited microorganisms contribute to cell wall repair indirectly by modulating plant hormone balance or providing precursors for cell wall biosynthesis, thereby preserving the structural integrity essential for nutrient acquisition (Ma et al. 2024).

Biotic stress, particularly from pathogenic microbes, fundamentally reshapes the RMCE. Far from being isolated invaders, pathogens are active players that utilize complex biochemical arsenals to manipulate both the host plant and the broader rhizosphere community. Fungal pathogens, for instance, deploy nonproteinaceous chemical effectors, such as fungal secondary metabolites (FSMs), which act as “social influencers” within the rhizosphere (Rangel et al. 2021). FSMs can actively suppress host immunity while simultaneously altering the surrounding bacterial and fungal landscape, either by inhibiting competing beneficial commensals (e.g. through antimicrobial mycotoxins) or by facilitating alliances with specific co-habitating microbes to promote their own niche establishment (Rangel et al. 2021). To counter these invasions, plants mount a “cry-for-help” response, dynamically adjusting their root exudate chemistry (e.g. secreting malate or specific phenolics) to selectively recruit suppressive microbial consortia that physically shield the roots and synthesize defensive antibiotics (Yuan et al. 2018), thereby attempting to restore RMCE equilibrium.

These adaptive strategies, however, involve significant trade-offs. Allocating photosynthate to defensive compounds (e.g. coumarins and phenolics) or to sustaining specialized microbial partners reduces the carbon available for biomass production and yield (Sorty et al. 2025). We hypothesize that adaptive restoration is achieved through dynamic rewiring of rhizosphere metabolic networks rather than isolated stress-response pathways. Under chronic stress, this diversion can impose a persistent energetic burden and constrain agronomic performance. Therefore, future agricultural strategies must aim to prime or augment these natural circuit-repair mechanisms, through targeted microbial inoculants, soil amendments, or breeding for optimized exudation profiles, without exacerbating carbon trade-offs. Ultimately, adaptive restoration enables rhizosphere systems to recover RMCE following environmental disturbance.

### Toward a reinforcing RMCE framework

Collectively, these four ecological processes establish a reinforcing RMCE framework that explains how rhizosphere metabolic circularity is generated, maintained over time, organized across space, and restored following environmental disturbance (Fig. 1). Rather than representing independent mechanisms, these dimensions operate as an integrated ecological cycle in which each process reinforces the next. (i) Metabolic generation. Reciprocal plant−microbe metabolic dialogues initiate the resource cycle through root exudation and microbial nutrient mobilization (Korenblum et al. 2022). This baseline generation is further fine-tuned by multitiered cross-kingdom signaling that coordinate plant physiology with microbial activity (Ortiz-Castro and López-Bucio 2019). (ii) Metabolic persistence. The live-dead plant−microbe continuum functions as the long-term storage vault of the RMCE. It converts transient biomass into persistent microbial necromass and slow-cycling MAOM, physically and chemically buffering the soil nutrient supply against environmental fluctuations over ecological timescales (Li et al. 2024). (iii) Spatial organization. The rhizosphere is organized into distinct subzones that establish microscale gradients, rather than functioning as a homogenous matrix (Mueller et al. 2024). Spatial heterogeneity provides the structural blueprints that maximizes RMCE efficiency by driving niche partitioning, reducing direct competitive overlap, and facilitating complex metabolic cross-feeding. (iv) Adaptive restoration. Adaptive resilience serves as the dynamic circuit-repair mechanism that maintains RMCE equilibrium during environmental perturbations. Under abiotic or biotic stressors, plants dynamically reprogram their exudation profiles to recruit stress-tolerant microbial consortia (Ling et al. 2023), or mounts a metabolic “cry-for-help” to selectively enrich suppressive microbiomes and restore functional stability (Yuan et al. 2018).

Together, metabolic generation fuels resource exchange, metabolic persistence sustains carbon and nutrient cycling across the live-dead continuum, spatial organization optimizes microscale resource allocation, and adaptive restoration re-establishes disrupted metabolic networks. Their continuous interaction forms a reinforcing RMCE framework that links temporal persistence, spatial organization, and adaptive recovery into a unified theory of rhizosphere metabolic circularity.

## Translating the RMCE framework into scenario-based management

Translating the RMCE framework into field practice requires management strategies that directly target the four interconnected dimensions of RMCE: metabolic generation, metabolic persistence, spatial organization, and adaptive restoration. Rather than applying universal interventions, RMCE provides a scenario-based decision framework in which management inputs are selected according to the dominant ecological bottlenecks limiting rhizosphere metabolic circularity. By manipulating root exudation, microbial necromass turnover, rhizosphere spatial architecture, and adaptive cross-kingdom signaling, scenario-based interventions can be tailored to specific environmental and agronomic constraints.

In nutrient-deficient soils, the primary objective is to maximize nutrient mobilization while minimizing carbon loss from the rhizosphere. The RMCE framework predicts that plants optimize carbon allocation by secreting exudates that recruit microorganisms with complementary nutrient-acquisition traits. Precision plant breeding and genome editing (e.g. CRISPR−Cas9) should prioritize exudation traits that enhance the release of specific functional metabolites, such as coumarins for iron chelation or flavonoids for recruiting nitrogen-fixing and phosphorus-solubilizing microorganisms (Suresh et al. 2025). Rather than constitutively increasing total exudation, breeding for exudation plasticity—the capacity to dynamically alter exudate profiles in response to nutrient availability—improves nutrient-use efficiency while conserving photosynthates. Microbial inoculants should likewise be designed as functionally complementary consortia rather than collections of plant-growth-promoting strains. Combining nutrient-solubilizing bacteria, mycorrhizal fungi, and saprophytic decomposers that recycle microbial necromass enhances immediate nutrient acquisition while sustaining long-term regeneration within the RMCE loop (Khan 2022). Timing is also critical, inoculation during peak exudation stages (e.g. early growth or flowering) maximizes colonization and functional integration (Moreno et al. 2025).

In compacted, eroded, or intensively managed systems, the major constraint is often the disruption of rhizosphere spatial organization rather than nutrient availability alone. Management should focus on restoring microscale habitat heterogeneity to re-establish spatial subzones, microbial niche complementarity, and streamlined cross-feeding (Korenblum et al. 2022). Practices such as conservation tillage, diversified crop rotations, intercropping, cover cropping, and localized nutrient placement reconstruct heterogeneous soil architecture that preserves microscale metabolite gradients. Organic amendments (e.g. compost and biochar) further promote aggregate formation, protect microbial necromass, and improve the physical stability of metabolic hotspots (Wang and Kuzyakov 2023). Reducing broad-spectrum pesticide inputs is equally important because preserving cross-kingdom signaling networks allows plants and microorganisms to maintain coordinated metabolic regulation under changing environmental conditions (Korenblum et al. 2022).

Under drought, salinity, thermal stress, or pathogen attack, sustaining the RMCE depends on preserving adaptive metabolic exchange rather than maximizing yield potential. Interventions should prioritize crop varieties capable of dynamic exudate reprogramming under stress to recruit beneficial microbes that mitigate oxidative damage, improve osmotic adjustment, and regulate stress hormones (e.g. ACC deaminase producers) (Moon and Ali 2022). In monoculture systems plagued by autotoxicity, introducing specialized bacterial consortia capable of enzymatically degrading phenolic autotoxins (e.g. p-coumaric or ferulic acids) restores rhizosphere health (Schaefer et al. 2008). Under pathogen threats, applying exogenous, nontoxic metabolic triggers (e.g. trace concentrations of specific malate isomers or quorum-sensing CDPs) artificially primes the rhizosphere “cry-for-help” response (Yuan et al. 2018, Chen et al. 2023). This artificial priming simulates a pathogen invasion, recruiting indigenous antibiotic-producing *Streptomyces* and *Bacillus* strains to form protective bio-barriers without imposing significant yield trade-offs (Korenblum et al. 2022).

Collectively, these scenario-based applications demonstrate that RMCE is not a collection of isolated agronomic practices, but a predictive decision framework in which management interventions are matched to the dominant ecological processes limiting rhizosphere metabolic circularity.

## Challenges and future directions

Despite substantial progress in understanding RMCE, translating this framework into scalable and sustainable agricultural practices remains challenging. Future research should focus on four priorities: quantifying metabolic persistence across the live-dead continuum, resolving the spatial organization of rhizosphere metabolic interactions, predicting adaptive restoration under environmental change, and bridging the gap between mechanistic understanding and field implementation.

### Quantifying metabolic persistence across the live-dead continuum

Although microbial necromass is recognized as a principal contributor to soil carbon storage, its role as an active component of RMCE remains largely conceptual. Future studies should quantify how necromass production, decomposition, stabilization, and reutilization are coupled with root exudation dynamics across different environmental conditions. Integrating stable isotope probing (^13^C and ^15^ N), metabolomics, and necromass biomarkers into quantitative carbon-flow models will enable direct estimation of CUE and nutrient recycling rates within RMCE networks (Tan et al. 2026).

### Resolving the spatial organization of rhizosphere metabolic interactions

Current approaches rarely capture the spatial distribution of metabolites, microorganisms, and necromass simultaneously, limiting our ability to identify functional hotspots within the rhizosphere. Emerging technologies, including imaging mass spectrometry, microfluidic rhizosphere-on-a-chip platforms, advanced spectroscopy, and spatial multiomics, offer opportunities to resolve spatially structured metabolic interactions from single-cell to aggregate scales. Emerging sensing, imaging, and computational tools are enabling nano-to-macroscale rhizosphere observation, linking local root−soil interactions to ecosystem-scale carbon and climate predictions (Ahkami et al. 2024). These empirical tools will allow direct testing of whether microscale spatial heterogeneity optimizes RMCE efficiency by maximizing niche complementarity and suppressing competitive interference.

### Predicting adaptive restoration under environmental change

Plants and microorganisms continuously adjust metabolic investments under environmental stress (e.g. drought, nutrient limitation, salinity, and other stresses), yet the metabolic costs and yield trade-offs governing these adaptive responses remain poorly understood. Future studies should combine long-term field experiments with systems biology and machine learning to determine how plants and microorganisms dynamically restore metabolic circularity following environmental perturbations, while minimizing the carbon costs associated with stress adaptation.

### Bridging the lab-to-field gap

The most fundamental barrier lies in the gap between controlled lab/greenhouse experiments and real-world filed applications. Field conditions introduce complex soil heterogeneity, weather fluctuations, and operational constraints. Long-term, multisite experiments that simultaneously manipulate plant genotype, microbial consortia, and soil management practices are therefore essential for validating RMCE predictions across diverse agroecosystems. Establishing standardized RMCE performance metrics that integrate yield stability, nutrient-use efficiency, microbial necromass accumulation, and soil carbon sequestration will be essential for evaluating management effectiveness across agroecosystems.

## Conclusion

This perspective expands the traditional RMCE framework by integrating metabolic persistence, spatial organization, and adaptive restoration into a unified theory of rhizosphere metabolic circularity. While intensive agricultural practices have historically disrupted RMCE balance, targeted management strategies that focus on exudate quality, necromass dynamics, spatial architecture, and signal crosstalk will provide a framework for ecosystem restoration. By integrating mechanistic understanding with scenario-based management, RMCE offers a scalable foundation for designing resilient agroecosystems that simultaneously enhance crop productivity, promote long-term soil carbon sequestration, and strengthen ecosystem resilience in a changing world.

## Data Availability

No new data were generated or analyzed in support of this article.
